# BmK AEP, an Anti-Epileptic Peptide Distinctly Affects the Gating of Brain Subtypes of Voltage-Gated Sodium Channels

**DOI:** 10.3390/ijms20030729

**Published:** 2019-02-08

**Authors:** Fan Zhang, Ying Wu, Xiaohan Zou, Qinglian Tang, Fang Zhao, Zhengyu Cao

**Affiliations:** State Key Laboratory of Natural Medicines and Jiangsu Provincial Key Laboratory for TCM Evaluation and Translational Development, School of Traditional Chinese Pharmacy, China Pharmaceutical University, Nanjing 211198, China; zhangfan20111112@126.com (F.Z.); wingwycpu@126.com (Y.W.); imzouxiaohan@163.com (X.Z.); cputangqinglian@163.com (Q.T.); zhaofang180930@163.com (F.Z.)

**Keywords:** BmK AEP, anti-epilepsy, action potentials, voltage-gated sodium channels

## Abstract

BmK AEP, a scorpion peptide purified form the venom of *Buthus martensii* Karsch, has been reported to display anti-epileptic activity. Voltage-gated sodium channels (VGSCs) are responsible for the rising phase of action potentials (APs) in neurons and, therefore, controlling neuronal excitability. To elucidate the potential molecular mechanisms responsible for its anti-epileptic activity, we examined the influence of BmK AEP on AP firing in cortical neurons and how BmK AEP influences brain subtypes of VGSCs (Na_v_1.1–1.3 and Na_v_1.6). BmK AEP concentration-dependently suppresses neuronal excitability (AP firing) in primary cultured cortical neurons. Consistent with its inhibitory effect on AP generation, BmK AEP inhibits Na^+^ peak current in cortical neurons with an IC_50_ value of 2.12 µM by shifting the half-maximal voltage of activation of VGSC to hyperpolarized direction by ~7.83 mV without affecting the steady-state inactivation. Similar to its action on Na^+^ currents in cortical neurons, BmK AEP concentration-dependently suppresses the Na^+^ currents of Na_v_1.1, Na_v_1.3, and Na_v_1.6, which were heterologously expressed in HEK-293 cells, with IC_50_ values of 3.20, 1.46, and 0.39 µM with maximum inhibition of 82%, 56%, and 93%, respectively. BmK AEP shifts the voltage-dependent activation in the hyperpolarized direction by ~15.60 mV, ~9.97 mV, and ~6.73 mV in Na_v_1.1, Na_v_1.3, and Na_v_1.6, respectively, with minimal effect on steady-state inactivation. In contrast, BmK AEP minimally suppresses Na_v_1.2 currents (~15%) but delays the inactivation of the channel with an IC_50_ value of 1.69 µM. Considered together, these data demonstrate that BmK AEP is a relatively selective Na_v_1.6 gating modifier which distinctly affects the gating of brain subtypes of VGSCs.

## 1. Introduction

Scorpion venoms are rich sources of bioactive peptides that modulate ion channel and receptor activities by modulation of channel gating kinetics [1,2,3,4]. These peptides provide invaluable pharmacological probes in exploring the structure and function of ion channels due to their exquisite selectivity [2,3,5]. They have displayed the potential to treat cancer [6] and neuronal [7], autoimmune [8], and cardiovascular diseases [9]. In general, according to the peptide length, scorpion toxins are classified into long-chain toxins and short-chain toxins. The long-chain scorpion toxins composed of 58–76 amino acid residues mainly act on voltage-gated sodium channels (VGSCs) while the short-chain scorpion toxins containing 30–40 amino acid residues generally target K^+^ or Cl^−^ channels [5,10,11]. The long-chain scorpion toxins can be categorized into excitatory mammalian toxins and depressant insect toxins based on their activity in mammals and insects [12].

The scorpion *Buthus martensii* Karsch (BmK) has been effectively used in traditional Chinese medicine for alleviating pain, epilepsy, and facial paralysis [13]. BmK AEP, a scorpion toxin identified from *B. martensii* Karsch, has been reported to display anti-epileptic activity in a coriaria lactone-induced epileptic model in rat without mammalian toxicity [14]. Structurally, BmK AEP shares high homology with depressant insect scorpion toxins, such as BmK ITa, BmK ITb [15], BmK IM [16], and LqqIT2 [17]. Similar to BmK AEP, the recombinant BmK IM also displays anti-epileptic activity in the pentylenetetrazol-induced seizure rat model [18]. The depolarized shift of activation voltage on Na^+^ currents in hippocampal neurons was proposed to be responsible for the anti-epileptic effect of BmK IM [18]. In addition, scorpion stings may cause epilepsy, especially children with immature blood-brain barrier, possibly because α-scorpion toxins induce increments in action potential firing and sympathetic excitation [19,20,21].

VGSCs play a crucial role in generating the rising phase of action potentials (APs) in neurons and, therefore, in controlling neuronal excitability. According to the sequence homology of the α-subunits, VGSCs can be categorized into nine subtypes (Na_v_1.1–1.9) with relatively tissue-specific expression [22]. Among the nine VGSC subtypes, Na_v_1.1–1.3 and Na_v_1.6 are predominantly expressed in the brain [23]. Many studies have demonstrated that channelopathy in VGSCs is one of the crucial reasons contributing to epilepsy [23,24], and clinic drugs targeting to VGSCs (such as carbamazepine) display therapeutic efficacy against seizures [25,26].

In this study, we therefore examined the influence of BmK AEP on neuronal excitability and how BmK AEP influences brain VGSC subtypes (Na_v_1.1–1.3 and Na_v_1.6). We demonstrate that BmK AEP suppresses AP firing in primary cultured cortical neurons and inhibits Na^+^ peak current in cortical neurons. Similar to its action on Na^+^ currents elicited in cortical neurons, BmK AEP suppresses the Na^+^ currents of Na_v_1.1, Na_v_1.3, and Na_v_1.6 with IC_50_ values of 3.20, 1.46, and 0.39 µM, respectively, suggesting that BmK AEP is a relatively selective Na_v_1.6 gating modifier. In contrast, BmK AEP minimally suppresses the peak currents of Na_v_1.2 but inhibits the inactivation of Na_v_1.2. Considered together, our data demonstrate that BmK AEP represents a unique toxin that distinctly affects the gating properties of brain VGSC subtypes.

## 2. Results

### 2.1. Influence of BmK AEP on Action Potential Firing in Cerebral Cortical Neurons

**Figure 1:**
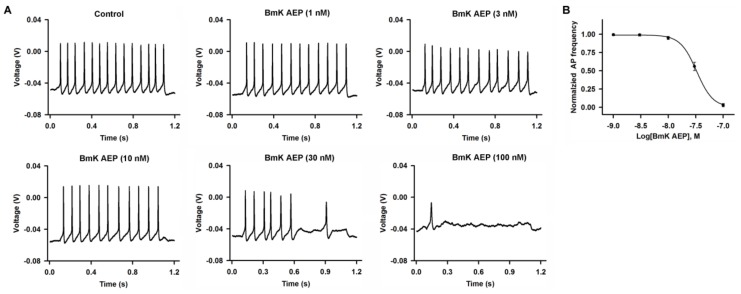
Influence of *Buthus martensii* Karsch (BmK) AEP on action potential firing in primary cultured mouse cortical neurons. (**A**) Representative traces of action potential firing evoked by a 30-pA current stimulation for 1000 ms in the presence of different concentrations of BmK AEP. (**B**) Concentration–response relationship of BmK AEP inhibition of action potential frequency. Each data point represents the mean ± SEM (*n* = 5–7).

BmK AEP has been shown to display anti-epileptic activity in a coriaria lactone-induced epileptic model in rat [14]. We therefore evaluated the effect of BmK AEP on neuronal excitability in primary cultured cortical neurons. The APs were elicited by injection of a 30-pA current. Bath application of BmK AEP concentration-dependently suppressed the AP firing frequency (Figure 1). At concentrations of 3 and 10 nM, BmK AEP displayed negligible effects on AP firing. However, at higher concentrations (30 and 100 nM), BmK AEP significantly suppressed AP firing. The IC_50_ value for BmK AEP suppression of AP firing is 33.1 nM (95% confidence interval (95% CI): 21.6–57.2 nM).

### 2.2. Influence of BmK AEP on VGSCs in Primary Cultured Cortical Neurons

**Figure 2:**
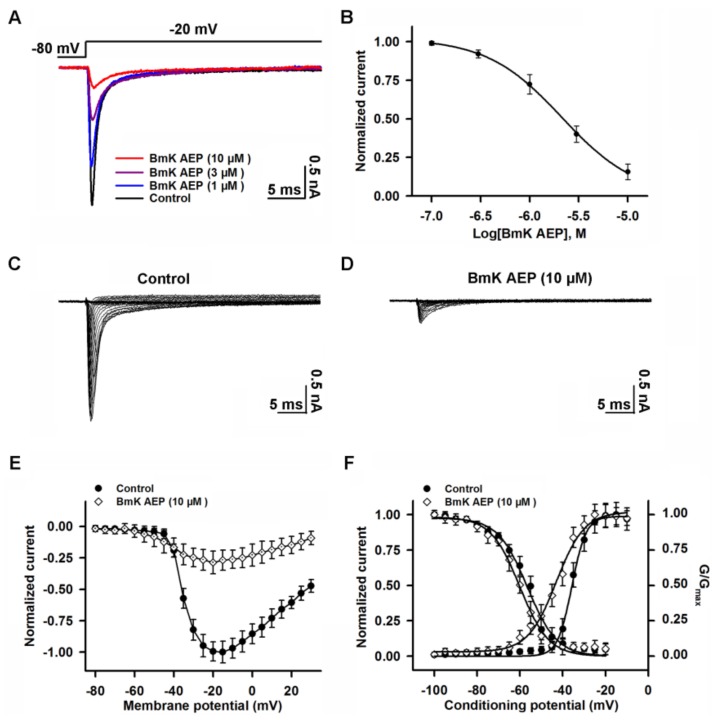
Influence of BmK AEP on voltage-gated sodium channel (VGSC) currents in primary cultured mouse cortical neurons. (**A**) BmK AEP inhibited the VGSC currents. Na^+^ currents were elicited by a 50-ms depolarization to −20 mV from the holding potential of −80 mV. (**B**) Concentration–response relationship of BmK AEP inhibition of Na^+^ currents. (**C**,**D**) Representative traces of Na^+^ currents in the absence and presence of 10 µM BmK AEP, respectively. Currents were triggered by 50-ms depolarizations from −100 to 30 mV in a 5-mV step. (**E**) Normalized peak current–membrane voltage (I–V) relationship of Na^+^ currents in the absence and presence of 10 µM BmK AEP. (**F**) Effect of BmK AEP (10 µM) on the steady-state activation and inactivation of VGSCs. Each data point represents the mean ± SEM (*n* = 5–8).

VGSCs are responsible for initiating APs in brain neurons. To investigate the molecular targets and mechanism of BmK AEP, we evaluated the influence of BmK AEP on VGSC currents in primary cultured cortical neurons. VGSC currents were elicited by a 50-ms pulse depolarized from a holding potential of −80 to −20 mV. Bath application of BmK AEP concentration-dependently inhibited VGSC peak currents in primary cultured cortical neurons with an IC_50_ value of 2.12 μM (1.25–3.76 µM, 95% CI) (Figure 2A,B). The maximal inhibition reached 85%. To test the influence of BmK AEP on VGSC activation, the Na^+^ currents were triggered by depolarized pulses from −100 mV to +30 mV in a 5-mV step in the absence and presence of BmK AEP (10 µM) (Figure 2C,D). At depolarization pulses below −40 mV, BmK AEP slightly increased the VGSC currents, whereas at the stronger depolarization potentials (˃ −40 mV), BmK AEP suppressed the Na^+^ peak currents (Figure 2E). BmK AEP shifted the voltage of half-maximal activation of VGSCs towards the hyperpolarized direction by ~7.83 mV (Figure 2F). BmK AEP minimally affected the steady-state inactivation of VGSCs (Figure 2F).

### 2.3. Influence of BmK AEP on hNa_v_1.1 Expressed in HEK-293 Cells

**Figure 3:**
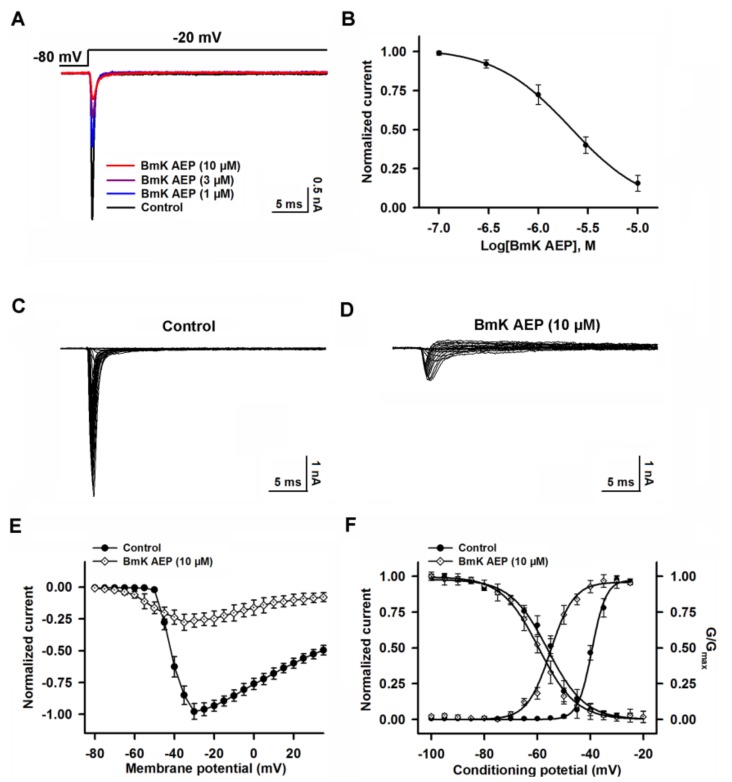
Influence of BmK AEP on hNa_v_1.1 expressed in HEK-293 cells. (**A**) BmK AEP suppressed the Na_v_1.1 peak current. Na_v_1.1 currents were elicited by a 50-ms depolarization to −20 mV from a holding potential of −80 mV. (**B**) Concentration–response relationships of BmK AEP inhibition on Na_v_1.1 currents. (**C**,**D**) Representative traces of Na_v_1.1 currents in the absence and presence of 10 µM BmK AEP, respectively. Currents were evoked by 50-ms depolarizations from −80 to 30 mV in a 5-mV step. (**E**) Normalized I–V relationship of Na_v_1.1 in the absence and presence of 10 µM BmK AEP. (**F**) Influence of BmK AEP (10 µM) on the steady-state activation and inactivation of Na_v_1.1. Each data point represents the mean ± SEM (*n* = 5–8).

Na_v_1.1 is the primary subtype expressed in cortical neurons and plays a crucial role in action potential firing in the CNS. We next evaluated the action of BmK AEP on brain VGSC subtypes. Bath application of BmK AEP suppressed Na_v_1.1 peak current evoked by a 50-ms depolarizing potential of −20 mV from a holding potential of −80 mV with an IC_50_ value of 3.20 µM (1.73–6.32 µM, 95% CI), and the maximal inhibition reached 82% (Figure 3A,B). Similar to the effect observed in cortical neurons, BmK AEP increased the Na_v_1.1 currents at the depolarization potentials below −45 mV, whereas at the stronger depolarization potentials (˃ −45 mV), BmK AEP suppressed the Na^+^ peak currents (Figure 3C–E). BmK AEP shifted the voltage of half-maximal activation of Na_v_1.1 towards the hyperpolarized direction by ~15.6 mV without affecting steady-state inactivation (Figure 3F).

### 2.4. Influence of BmK AEP on hNa_v_1.2 Expressed in HEK-293 Cells

**Figure 4:**
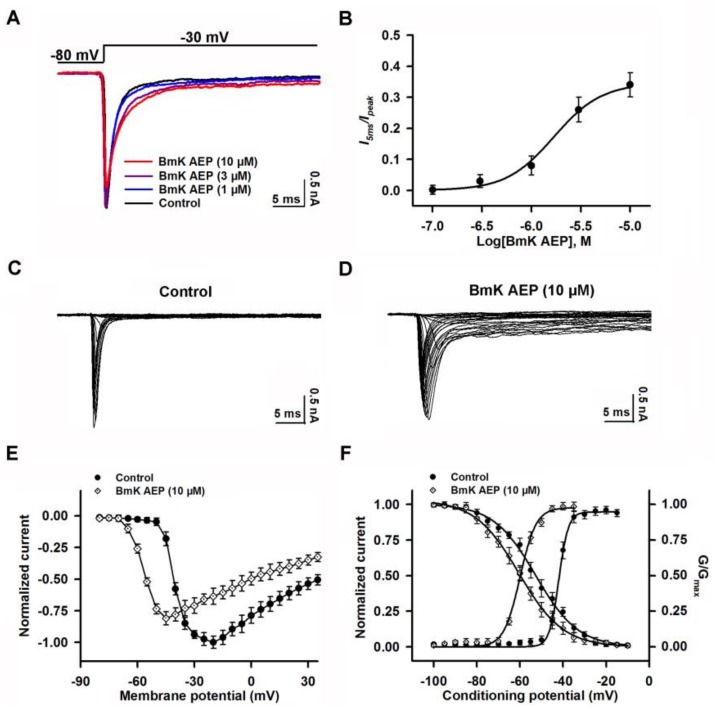
Influence of BmK AEP on hNa_v_1.2 expressed in HEK-293 cells. (**A**) BmK AEP delayed Na_v_1.2 channel inactivation. Na_v_1.2 currents were triggered by a 50-ms depolarization to −30 mV from a holding potential of −80 mV. (**B**) Concentration–response relationship of BmK AEP-delayed inactivation of Na_v_1.2 channels. (**C**,**D**) Representative traces of Nav1.2 currents in the absence and presence of 10 µM BmK AEP, respectively. Currents were triggered by 50-ms depolarizations from −100 to 30 mV in a 5-mV step. (**E**) Normalized I–V relationships of Na_v_1.2 currents in the absence and presence of 10 μM BmK AEP. (**F**) Effect of BmK AEP (10 µM) on the steady-state activation and inactivation of Nav1.2. Each data point represents the mean ± SEM (*n* = 5–8).

We next examined the effect of BmK AEP on Na_v_1.2 heterologously expressed in HEK-293 cells. In contrast to the significant effect on the suppression of Na_v_1.1peak current, bath application of 10 µM BmK AEP slightly suppressed the Na_v_1.2 peak current (~15%) (Figure 4A). However, BmK AEP significantly delayed Na_v_1.2 channel inactivation, and the EC_50_ value was calculated as 1.69 μM (0.93–2.76 µM, 95%CI) (Figure 4B). BmK AEP (10 µM) shifted both the voltages of half-maximal activation and steady-state inactivation towards the hyperpolarized direction by ~17.8 and ~7.7 mV, respectively (Figure 4C–F).

### 2.5. Influence of BmK AEP on hNa_v_1.3 Stably Expressed in HEK-293 Cells

**Figure 5:**
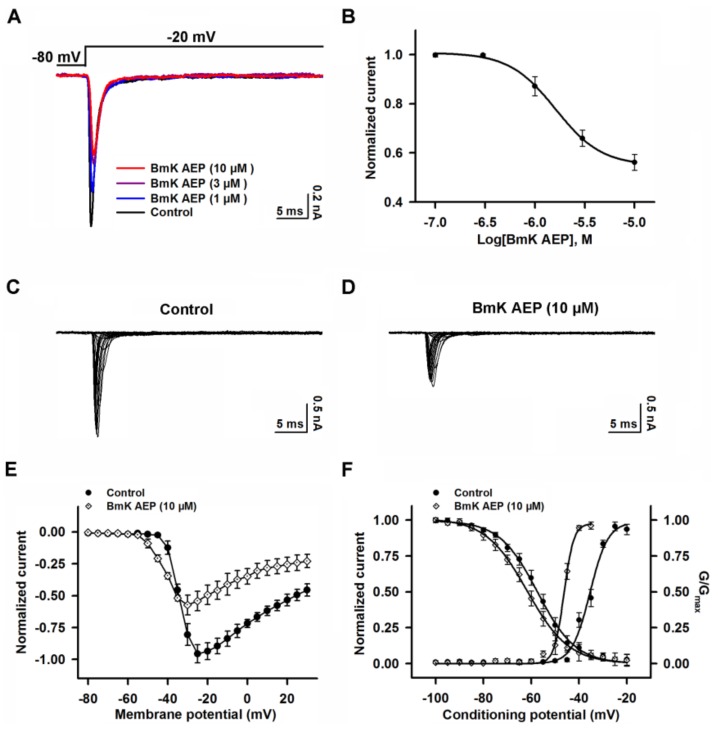
Influence of BmK AEP on hNa_v_1.3 expressed in HEK-293 cells. (**A**) Representative traces of BmK AEP suppression of the Na_v_1.3 peak current. Na^+^ currents were elicited by a 50-ms depolarization to −20 mV from a holding potential of −80 mV. (**B**) Concentration–response relationship curve of BmK AEP inhibition of Na_v_1.3 currents. (**C**,**D**) Representative traces of Na_v_1.3 currents in the absence and presence of 10 μM BmK AEP, respectively. Currents were trigged by 50-ms depolarizations from −100 to 30 mV in a 5-mV step. (**E**) Normalized I–V relationships of Na_v_1.3 currents in the absence and presence of 10 µM BmK AEP. (**F**) Effect of BmK AEP (10 µM) on the steady-state activation and inactivation of Na_v_1.3. Each data point represents the mean ± SEM (*n* = 5–8).

Similar to the effect on Na_v_1.1, bath application of BmK AEP suppressed the Na_v_1.3 peak current evoked by a 50-ms depolarizing potential of −20 mV from a holding potential of −80 mV with an IC_50_ value of 1.46 µM (0.91–2.56 µM, 95% CI) (Figure 5A,B). However, the maximal inhibition on Na_v_1.3 was only 56% (Figure 5B, Table 1). BmK AEP enhanced Na_v_1.3 currents at the depolarization potentials below −35 mV, whereas at the stronger depolarization potentials (˃ −35 mV), BmK AEP suppressed the Na^+^ peak currents (Figure 5C–E). BmK AEP shifted the voltage for half-maximal activation towards the hyperpolarized direction by ~10.0 mV while minimally affecting the steady-state inactivation of Na_v_1.3 (Figure 5F).

### 2.6. Influence of BmK AEP on hNa_v_1.6 Expressed in HEK-293 Cells

**Figure 6:**
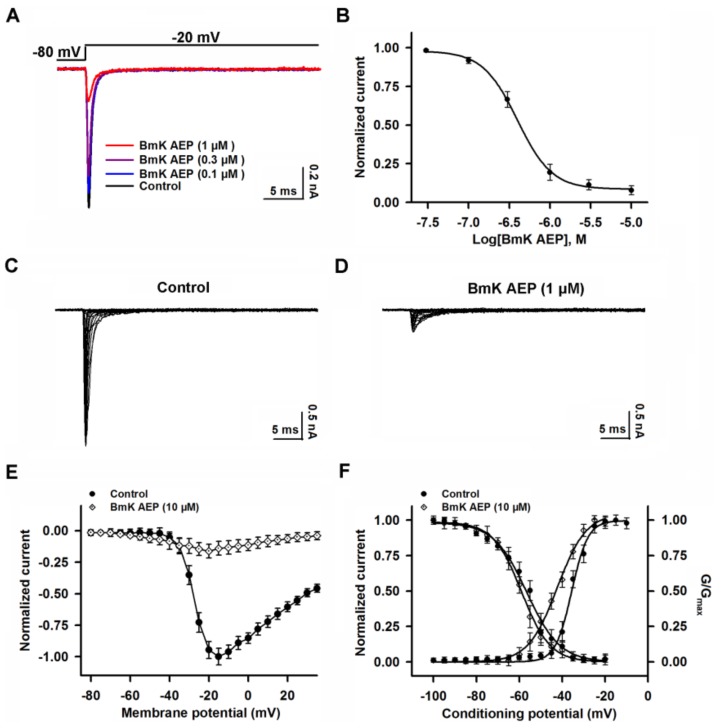
Influence of BmK AEP on hNa_v_1.6 expressed in HEK-293 cells. (**A**) BmK AEP suppressed the Na_v_1.6 peak current. Na^+^ currents were elicited by a 50-ms depolarization to −20 mV from a holding potential of −80 mV. (**B**) Concentration–response relationship curve of BmK AEP inhibition of Na_v_1.6 currents. (**C**,**D**) Representative traces of Na_v_1.6 currents in the absence and presence of 10 µM BmK AEP, respectively. Currents were trigged by 50-ms depolarizations from −80 to 30 mV in a 5-mV step. (**E**) Normalized I–V relationships of Na_v_1.6 currents in the absence and presence of 10 µM BmK AEP. (**F**) Effect of BmK AEP (10 µM) on the steady-state activation and inactivation of Na_v_1.6. Each data point represents the mean ± SEM (*n* = 5–7).

Na_v_1.6 was reported to be expressed in the brain [27]. The influence of BmK AEP on Na_v_1.6 was evaluated. Similar to the effect on Na_v_1.1, bath application of BmK AEP suppressed the Na_v_1.6 peak current evoked by a 50-ms depolarizing potential from a holding potential of −80 mV to −20 mV (Figure 6A,B). At concentrations greater than 3 µM, BmK AEP nearly completely suppressed Na_v_1.6 currents (Figure 6B). Non-linear regression revealed that the IC_50_ value for BmK AEP suppression of Na_v_1.6 current was 0.39 µM (0.26–0.57 µM, 95% CI) (Figure 6B, Table 1). BmK AEP slightly enhanced the Na_v_1.6 currents at the depolarization potentials below −35 mV, whereas at the stronger depolarization potentials (˃ −35 mV), BmK AEP suppressed the Na_v_1.6 peak currents (Figure 6C–E). BmK AEP shifted the voltage for half-maximal activation of Na_v_1.6 towards the hyperpolarized direction by ~6.7 mV without affecting the steady-state inactivation of Na_v_1.6 (Figure 6F).

## 3. Discussion

Scorpion toxins provide valuable tools to study the channel gating and function of a variety of ion channels/receptors [28]. BmK AEP has been reported to display anti-epileptic activity in a coriaria lactone-induced epileptic model in the rat with comparable efficacy to diazepam [14]. In this study, we demonstrated that BmK AEP suppressed AP firing and VGSC currents in cortical neurons consistent with its inhibitory effect on neuronal excitability. The IC_50_ value for BmK AEP suppression of AP firing is 33.1 nM, which is around 64-fold more potent than that on VGSC currents (2.12 µM). Whether the action on VGSC was responsible for its inhibitory effect on AP firing needs further examination. It should be noted that tetrodotoxin (TTX), a pore blocker of TTX-sensitive VGSCs, also suppressed the AP firing at ~90 times more potent than that on VGSC currents recorded in spinal cord neurons (Figure S1).

Long-chain scorpion toxins are generally recognized to be VGSC gating modifiers [29]. In general, these toxins can be categorized into α- and β-toxins based on their binding sites and actions on VGSCs [30,31]. α-Toxins bind to neurotoxin site 3 which is composed of the amino acid resides located in the S3-S4 loop and S1-S2 loop in domain IV of VGSCs; it delays the inactivation kinetics of the VGSCs by preventing the outward movement of the S4 segment of domain IV in response to depolarization [30,32]. β-Toxins bind to neurotoxin site 4 (S3–S4 of domain II) and shift the activation to negative potentials through a voltage sensor trapping mechanism [32]. We demonstrate that BmK AEP shifts the activation voltage to a hyperpolarized direction and represses the peak Na^+^ currents in primary cultured cortical neurons, two characteristics of β-toxin action on VGSCs. Structurally, BmK AEP is highly homologous (>83%) the scorpion insect β-toxins BmK ITa, BmK ITb, and LqqIT2 [15,17]. It has been demonstrated that BmK IM also displays anti-epileptic activity in the pentylenetetrazol-induced seizure model in rats by shifting the voltage for half-maximal activation towards the depolarized direction and suppressing Na^+^ currents [18]. These data imply that β-toxins may display anti-epileptic activity, although they distinctly modify the activation of VGSCs.

While BmK AEP shifts the activation voltage to more negative potentials in Na_v_1.1–1.3 and Na_v_1.6, which is consistent with β-toxin effects, it is also interesting that BmK AEP also delays channel inactivation of Na_v_1.2. These data suggest that in Na_v_1.2, BmK AEP displays both α-like and β-like properties. The IC_50_ value of BmK AEP on the suppression of Na^+^ currents of Na_v_1.6 is 0.39 µM, while in Na_v_1.1, and Na_v_1.3, the IC_50_ values are 3.20 and 1.46 µM, respectively. In addition to the potency difference, BmK AEP suppresses the Na^+^ currents of Na_v_1.1, Na_v_1.3, and Na_v_1.6 to distinct degrees with maximum inhibition of 82%, 56%, and 93%, respectively. Therefore, BmK AEP is a selective Na_v_1.6 gating modifier with 9.30 and 6.21-fold selectivity over Na_v_1.1, and Na_v_1.3, respectively. It is quite interesting that the degree of inhibition is inversely correlated with hyperpolarized shift of voltage for half-maximal activation (or maximum augmentation of Na^+^ currents at relatively less depolarized potentials in Na_v_1.1–1.3 and Na_v_1.6).

Sodium channel blockers, including phenytoin, carbamazepine, lamotrigine, oxcarbazepine, rufinamide, and lacosamide, represent the main anti-epileptic drugs [33]. Generally, these classical sodium channel blocking drugs are nonselective for different subtypes of VGSCs (Na_v_1.1–1.7). These nonselective inhibitors are commonly used for controlling partial seizures and many different causes of seizures. According to the drug-receptor hypothesis, the varying ability of these drugs to treat different epilepsy diseases depends on their binding and action [34]. In the present study, BmK AEP differentially suppressed the peak current of brain sodium channel subtypes (Na_v_1.1, 1.3, and Na_v_1.6) with β-toxin effects (binding to the S3–S4 of domain II of VGSC), except for the Na_v_1.2 channel. The inhibition of the peak current of sodium channels in the cortical neurons by BmK AEP resulted in the suppression of APs and consequently blocked hyperexcitability in brain neurons, therefore contributing to the anti-epileptic activity of BmK AEP. However, the nonselective sodium channel anti-epileptic drugs affecting the interactions of the voltage-gated sodium channels are still lacking, which could be due to the difficulty of the research. Further studies are needed to reveal how BmK AEP affects the interactions of the brain sodium channel subtypes. It is noteworthy that BmK AEP may have problems in crossing blood-brain barrier, which leads to its increase in concentration in the blood, which will result in BmK AEP targeting VGSCs in the peripheral nervous system and therefore causing potential adverse effects.

In summary, in the present study, we demonstrated that BmK AEP suppresses neuronal excitability and displays β-toxin effects in in cortical neurons. BmK AEP selectively suppresses Na_v_1.6 currents when compared with other brain subtypes of VGSCs. An interesting feature of BmK AEP is that although BmK displays β-toxin effects on Na_v_1.1, Na_v_1.3, and Na_v_1.6, BmK AEP also delays the inactivation kinetics of Na_v_1.2. These data suggest that BmK AEP may present a useful tool to investigate the gating mechanism of VGSCs.

## 4. Materials and Methods

### 4.1. Materials and Animals

Lyophilized crude venom of *B. martensii* Karsch was purchased from a domesticated scorpion farm (Zhengzhou, Henan, China) and was the same source as described previously [35]. Sephadex G-50 and CM-Sephadex C-50 resins were obtained from Pharmacia Fine Chemicals (Uppsala, Sweden). Fetal bovine serum (FBS), trypsin, and l-glutamine were obtained from Thermo Fisher Scientific (Grand Island, NY, USA). Trifluoroacetic acid and inorganic salts were obtained from Sigma-Aldrich (St. Louis, MO, USA). HEK-293 cells stably expressing hNa_v_1.1, hNa_v_1.3, or hNa_v_1.6 were generous gifts from Christoph Lossin (University of California, Davis, CA, USA) and are the same cells used in the previous study [36]. HEK-293 cells stably expressing Na_v_1.2 was gifted by Yuan Chen (Zhejiang Agriculture & Forestry University, Deqing, China) as described previously [37]. C57BL/6J mice were purchased from the Model Animal Research Center of Yangzhou University (Yangzhou, China).

### 4.2. BmK AEP Purification

The BmK crude venom was dissolved in ddH_2_O and centrifuged to remove the sediment. After gel filtration in a Sephadex G-50 column, 4 fractions (G1–G4) were collected. These fractions were subjected to an LC-MS analysis to identify the BmK AEP. In the G-2 fraction, a peptide with molecular weight of 6730.3 Da close to that of BmK AEP (6730.4 Da) was found. The fraction was then subjected to HPLC purification, and the targeted toxin was collected and lyophilized. To further confirm the identity, the purified toxin was subjected to the Edman degradation and the first 15 amino acid residues in N-terminal was determined to be DGYIRGSNGCKVSCL, which was identical to that of BmK AEP. In addition, after reduction and *S*-carboxymethylation, the toxin was cleaved by *Staphylococcus aureaus* V_8_ protease or Lysyl Endopeptidase and the enzymatic fragments were subjected to LC-MS analysis. After *S. aureaus* V_8_ protease cleavage, three fragments with molecular weights of 607.4, 1275.5, and 537.2 Da corresponding to the molecular weights of fragments containing residues of 20–24, 46–56, and 47–61 of BmK AEP, respectively, were observed. Similarly, four fragments with molecular weights of 1226.5, 2056.5, 1460.6, and 434.2 Da corresponding to the molecular weights of fragments containing residues of 1–11, 24–39, 40–51, and 52–54 of BmK AEP, respectively, were observed. Together, the purified toxin has the same sequence of BmK AEP.

### 4.3. Primary Cultures of Cortical Neurons

Animal experimentation protocols (81473539) were approved (12-05-2017) by the Animal Experimentation Ethics Committee of China Pharmaceutical University. All experiments conformed to the rule of minimizing animal suffering and numbers. The cortical neurons were dissociated from the cortex of C57BL/6 mice (embryonic day 16 of either gender) as described previously and maintained in Neurobasal complete medium (Neurobasal medium + 2% NS21, 1 mM l-glutamine, and 1% HEPES) containing 5% FBS [35]. The dissociated cortical neurons were plated onto poly-l-lysine-coated (50 µg/mL) 35-mm diameter dishes (Corning Incorporation, Corning, NY, USA) at a density of 1.5 × 10^4^ cells/dish. The culture medium was half-replaced by Neurobasal complete medium (serum free) at days in vitro (DIV) 5 and 7. The neurons were maintained at standard conditions (5% CO_2_, 95% humidity at 37 °C) and were used at DIV 8.

### 4.4. Culture of HEK-293 Cells Heterologously Expressing VGSC Subtypes

HEK-293 cells stably expressing individual VGSC subtypes were maintained in a culture medium (DMEM supplemented with 10% FBS, 500 µg/mL G-418, 100 units/mL penicillin, and 0.1 mg/mL streptomycin) in poly-d-lysine (10 µg/mL) pre-coated T75 flasks (Corning Incorporation, Corning, NY, USA). At around 70% confluency, cells were digested with 0.05% trypsin and plated into a poly-d-lysine pre-coated 35-mm diameter dish (Corning Life Sciences, Acton, MA, USA) at a density of 1.5 × 10^4^ cells/dish. Cells were maintained at standard conditions (5% CO_2_, 95% humidity at 37 °C) for 24 h before the experiment.

### 4.5. Voltage-Clamp and Current-Clamp Electrophysiology

Whole cell patch clamp was used to record the voltage-gated sodium currents in mouse cortical neurons or HEK-293 cells expressing VGSC subtypes using an EPC-10 amplifier (HEKA Electronics, Lambrecht, Germany) as described previously [5]. Fire-polished electrodes were fabricated from 1.5-mm glass capillaries using a horizontal micropipette puller (P-1000, Sutter Instrument Company, Novato, CA, USA) with tip resistances of 2–3 MΩ. For recording Na^+^ currents in cortical neurons, the bathing solution was (in mM) 30 NaCl, 5 CsCl, 1.8 CaCl_2_, 5 KCl, 1 MgCl_2_, 25 d-glucose, 5 HEPES, and 90 tetraethylammonium-Cl (pH 7.4, adjusted with NaOH); and the pipette solution contained (in mM) 135 CsF, 10 NaCl, and 5 HEPES (pH 7.2, adjusted with CsOH). For recording Na^+^ currents in HEK-293 cells with VGSC subtype expression, the bathing solution contained (in mM) 130 NaCl, 1.5 CaCl_2_, 1.5 MgCl_2_, 4 KCl, 5 glucose, 5 HEPES, and 20 sucrose (pH 7.4, adjusted with NaOH); and the pipette solution was (in mM) 90 CsF, 60 CsCl, 10 NaCl, and 5 HEPES (pH 7.2, adjusted with CsOH). For current-clamp recording in the cortical neurons, fire-polished electrodes (3.0–5.0 MΩ) were filled with a pipette solution that was (in mM) 140 KCl, 5 MgCl_2_, 2.5 CaCl_2_, 5 EGTA, 4 ATP, 0.3 GTP, and 10 HEPES (pH 7.3, adjusted with KOH); and neurons were bathed in a bathing solution containing (in mM) 140 NaCl, 1 MgCl_2_, 5 KCl, 2 CaCl_2_, 10HEPES, and 10 glucose (pH 7.3, adjusted with NaOH). To study the effect of BmK AEP (10 µM) on peak current-membrane voltage (I–V) relationships, the Na^+^ currents were triggered by depolarized pulses from −100 mV to +30 mV in a 5-mV step. The steady-state inactivation was tested by a standard double pulse protocol in which a series of pre-pulses with potentials ranging from −130 mV to +20 mV with a 5-mV increment were applied for 500-ms in the absence and presence of 10 µM BmK AEP before the Na^+^ current was triggered by a 50-ms depolarizing pulse to −20 or −30 mV. Concentration–response curves of peptides on the channels were fitted by the Hill equation as follows: I_nor_= C + A/[1 + ([BmK AEP]/IC_50_)*^p^*], where I_nor_, V_1/2_, and *p* are the normalized peak current, the half-maximal inhibitory concentration, and the slop factor, respectively. The steady-state inactivation curve was fitted by the Boltzmann equation as follows: [I/I_max_ = 1/(1 + exp(V_1/2_-V)/*k*)], where V, V_1/2_, and *k* are the membrane potential of the conditioning step, the membrane potential at half-maximal inactivation, and the slope factor for the inactivation curve, respectively. All data points represent the means ± SEM.

## Figures and Tables

**Table 1:** The potency and maximum inhibition of BmK AEP in brain subtypes of VGSCs.

VGSC Subtypes	Influence	Maximum Inhibition/Delay (%)	IC_50_ (μM)^a^ 95% CI	Selectivity for Na_v_1.6
Na_v_1.1	Inhibition of peak current	82%	3.20 (1.73–6.32)	9.30
Na_v_1.2	Delay of inactivation	34%	1.69 (0.93–2.76)	-
Na_v_1.3	Inhibition of peak current	56%	1.46 (0.91–2.56)	6.21
Na_v_1.6	Inhibition of peak current	93%	0.39 (0.26–0.57)	1

Selectivity = (MaxNa_v_1.6/MaxNa_v_1.x) × (IC_50_Na_v_1.x/IC_50_Na_v_1.6); ^a^, IC_50_ values were measured at 10 Hz; “–” represents no comparison with Na_v_1.6; data are shown as means ± SEM; *n* = 5–8 for each subtype.

## Supplementary Materials

Supplementary materials can be found at www.mdpi.com/xxx/s1.

## Abbreviations

APs	Action potentials
BmK	*Buthus martensii* Karsch
CI	Confidence Interval
DIV	Days in vitro
FBS	Fetal bovine serum
I-V	Current-membrane voltage
VGSCs	Voltage-gated sodium channels
