# Supplementary material for: BmK AEP, an Anti-Epileptic Peptide Distinctly Affects the Gating of Brain Subtypes of Voltage-Gated Sodium Channels
Source: Int J Mol Sci. 2019 Feb 8;20(3):729. doi: 10.3390/ijms20030729 (PMC6387193; doi:10.3390/ijms20030729)
Supplement: Supplementary file 1 [file ijms-20-00729-s001.pdf]

International Journal of
Molecular Sciences

MDPI

Supplementary Information

BmK AEP, an Anti-Epileptic Peptide Distinctly Affects the Gating of Brain Subtypes of Voltage-Gated Sodium Channels

Fan Zhang †, Ying Wu †, Xiaohan Zou, Qinglian Tang, Fang Zhao and Zhengyu Cao *

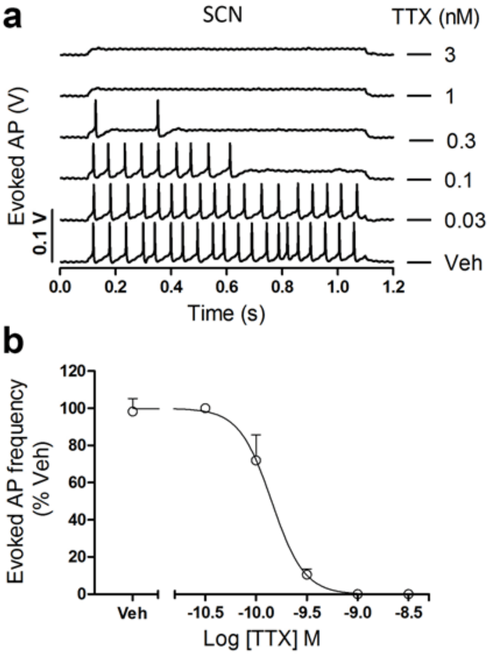

Figure 1. TTX blocks evoked action potentials in primary cultured spinal cord neurons. (A) Representative traces of APs evoked by an injection of 30-pA current in spinal cord neurons in the absence and presence of different concentrations of TTX as indicated. (B) Concentration-response relationship of TTX inhibition of evoked APs in spinal cord neurons. Each data point represents the mean ± SEM (*n* = 5).

© 2019 by the authors. Submitted for possible open access publication under the terms and conditions of the Creative Commons Attribution (CC BY) license (http://creativecommons.org/licenses/by/4.0/).
